# Osteoporosis independent of traditional risk factors in hyperthyroid patients in a resource-limited setting: An observational study

**DOI:** 10.1097/MD.0000000000048715

**Published:** 2026-05-08

**Authors:** Mahlet Tadesse, Brook Alemayehu Tesfaye, Bemnet Taye, Meti Wole, Mihiret Gebre, Sisaynesh Angota, Ahmed Reja

**Affiliations:** a Endocrinology and Metabolism Unit, Department of Internal Medicine, College of Health Sciences, Addis Ababa University, Addis Ababa, Ethiopia; b Department of Radiology, College of Health Sciences, Addis Ababa University, Addis Ababa, Ethiopia; c Endocrinology and Metabolism Unit, Department of Internal Medicine, College of Health Sciences, Rwanda University, Kigali, Rwanda; d Department of Internal Medicine, Eka Kotebe General Hospital, Addis Ababa, Ethiopia.

**Keywords:** bone mineral density, DXA, ethiopia, hyperthyroidism, osteoporosis, secondary osteoporosis

## Abstract

Hyperthyroidism accelerates bone turnover and increases the risk of secondary osteoporosis. However, evidence from sub-Saharan Africa remains scarce. This study assessed the prevalence and predictors of osteoporosis among hyperthyroid patients without traditional risk factors for bone loss at Tikur Anbessa Specialized Hospital, Addis Ababa, Ethiopia. A cross-sectional study was conducted from April to July/ 2025 among adults aged 18 to 50 years with confirmed hyperthyroidism at at least 6 months of follow-up. Patients with traditional osteoporosis risk factors, such as menopause, glucocorticoid use, rheumatoid arthritis, smoking were excluded. Socio-demographic clinical and biochemical data were collected. Bone mineral density at lumbar spine, total hip and femoral neck was measured using dual-energy X-ray absorptiometry (DXA). Multivariable logistic regression was used to identify predictors of osteoporosis. Among 51 participants (88.2% female; mean age 38.5 ± 7.4 years), 19 (37.3%) had DXA-confirmed osteoporosis. The median Z-score for lumbar, left total hip and left femoral neck were −1.5, −0.7, and −0.5, respectively. Osteoporosis was more common among patients with uncontrolled hyperthyroidism (66.7%) when compared to those with euthyroid (30.6%) and hypothyroid (33.3%) states. However, no independent predictors were identified on multivariable analysis. More than a third of hyperthyroid patients without traditional risk factors had low bone mineral density, underscoring the skeletal impact of thyroid hormone excess. Routine DXA screening and early intervention should be considered even for younger hyperthyroid patients in resource-limited settings.

## 1. Introduction

Hyperthyroidism affects approximately 0.7% to 1.4% of the global population, with a reported prevalence of 0.2% to 2.5% in iodine-sufficient regions.^[1]^ In Africa, rates of 2% and 1.6% have been reported in South Africa and Nigeria, respectively.^[2]^ A systematic review and meta-analysis of 17 studies estimated the prevalence of hyperthyroidism in Europe at 0.75%.^[3]^

Thyroid hormones are essential for skeletal growth and bone mass maintenance. Excess thyroid hormone accelerates bone turnover, leading to bone loss.^[4,5]^ Osteoporosis, the most common metabolic bone disease, is characterized by reduced bone mass and microarchitectural deterioration of bone tissue, resulting in impaired strength.^[6–8]^ Among the various risk factors for osteoporosis, hyperthyroidism is a recognized contributor.^[8]^

Hyperthyroidism shortens the normal bone remodeling cycle from approximately 200 to 100 days.^[9,10]^ This imbalance between bone resorption and formation causes an estimated 10% bone loss per remodeling cycle.^[9]^ Bone densitometry studies have demonstrated decreased bone mineral density (BMD) at all skeletal sites,^[3]^ with cortical bone more affected than trabecular bone.^[11]^

Despite global evidence, this relationship has not been fully explored in Ethiopia. Early diagnosis and treatment of osteoporosis in hyperthyroid patients are therefore critical to reducing morbidity and mortality.

## 2. Methods

### 2.1. Study design and setting

We conducted a cross-sectional study from April 1 to July 31, 2025 at TASH, the largest tertiary referral and teaching hospital in the country.

### 2.2. Study population

Adults aged 18 to 50 years diagnosed with hyperthyroidism who had follow-up for at least 6 months prior to the study and willing to give consent were included. Exclusion criteria include: known osteoporosis, history of prior pathologic fracture, post-menopausal women, women with premature ovarian failure, rheumatoid arthritis, glucocorticoid or other immunosuppressive use, smoking history and pregnancy.

### 2.3. Sample size

All consecutive eligible patients attending follow-up during the study period were enrolled.

### 2.4. Variables

The dependent variable was osteoporosis while independent variables included socio-demographic and economic factors (age, sex, educational level, marital status, residence, occupation, monthly income), disease related factors (causes of hyperthyroidism, duration of hyperthyroidism and anti-thyroid medication), biochemical factors [thyroid stimulating hormone, free tetraiodothyroxine (FT4), alkaline phosphatase (ALP), and and serum calcium (Ca)] and behavioral related factors (physical activity, cigarette smoking, dietary diversification, body mass index and adherence to medication).

### 2.5. Data collection and measurements

Socio-demographic and related data was collected via interview using an interviewer-administered structured questionnaire and supplemented by review of patient medical records. The questionnaire was developed in English, translated into Amharic and back-translated to English to ensure consistency. BMD was assessed using DXA scans of the lumbar spine, left total hip and left femoral neck and reported by a musculoskeletal radiologist. We used the DXA Z-score of <2.0 to define osteoporosis according to the International Society of Clinical Densitometry criteria.^[12]^

### 2.6. Data quality management

The data collection tool was pretested on 5 subjects (not part of the present study). Daily supervision, calibration of measuring device and cross-checks of questionnaires were performed to ensure data quality. Body weight was measured by calibrated digital weight scales. Standing height was taken in a standardized manner and the result rounded to the nearest 0.5 cm.

### 2.7. Statistical analysis

Data was double-checked for completeness and accuracy. It was then entered, cleaned, and analyzed using Statistical Package for the Social Sciences (SPSS) version 25. Continuous variables were summarized as mean (± standard deviation) if normally distributed and median [with inter-quartile ratio (IQR)] if not normally distributed. Categorical variable are described as frequency and percentages. Associations between osteoporosis and independent variables were assessed using bivariable and multivariable logistic regression analyses. Variables with *P* <.30 in bivariable analysis were included in the multivariable model. Adjusted odds ratio with a 95% CI were reported and statistical significance was defined as *P* <.05.

### 2.8. Ethical approval

Ethical clearance was obtained from the IRB of the department of Internal Medicine, College of health sciences, Addis Ababa University (Protocol No. 169/25). The study was conducted in accordance with the principles of the Declaration of Helsinki. Informed consent was collected from each study participant.

## 3. Result

### 3.1. Study participants

A total of 51 participants were analyzed. Majority of the patients (88 %) were female, had a mean age of 38.5 years while median duration of hyperthyroidism was 5 years. None of the patients were taking calcium or vitamin D supplements. Most (84.3%) patients were on anti-thyroid drug treatment with most taking propylthiouracil (PTU)- based regimen (Tables 1 and 2, Supplementary document).

**Table 1:** Socio-demographic and behavioral characteristics of the study population.

Variable	Categories	Number	Percent
Age (mean) in years		39.5 + 7.4	
Age categories (in years)	18–29	6	11.8
	30–39	15	29.4
	40–49	30	58.8
Sex	Male	6	11.8
	Female	45	88.2
BMI (mean) in kg/m^2^		26.1 + 5.4	
BMI categories in kg/m^2^	Underweight	5	9.8
	Normal	16	31.4
	Overweight	17	33.3
	Obesity	13	25.5
Marital status	Single	10	19.6
	Married	34	66.7
	Divorced or widowed	7	13.7
Educational status	No formal education	3	5.3
	Primary school	15	29.4
	Secondary school	18	35.3
	College level	15	29.4
Occupational status	Unemployed	28	54.9
	Private organization employee	9	17.6
	Government employee	8	15.7
	Others	6	11.8
Income in ETB-median (IQR)		4000 (0, 10,000)	
Income categories in ETB	=<2000 ETB	5	9.8
	2001–5000 ETB	6	11.8
	5001–1000 ETB	15	29.4
	>10,000 ETB	25	49.0
Alcohol intake*	Yes	6	11.8
	No	45	88.2
Dietary diversity score	0–3 food items	29	56.9
	4–6 food items	22	43.1
Tea consumption*	Yes	30	58.8
	No	21	41.2
Coffee consumption*	Yes	36	70.6
	No	15	29.4
Physical exercise*	Yes	19	37.3
	No	32	62.7

BMI = body mass index, ETB = ethiopian birr.

* See supplementary document for definitions.

**Table 2:** Disease related characteristics of the study population.

Variable	Categories	Frequency	Percent
Duration of hyperthyroidism (years)	Median (IQR)	5 (3, 11)	–
	< 5 yr	29	56.9
	> 5 yr	22	43.1
Cause of hyperthyroidism	TMNG or TA	19	37.3
	Grave Disease	32	62.7
Treatment status	On ATDs	43	84.3
	Off ATDs*	8	15.7
Type of treatment (n = 43)	PTU	34	79.1
	Carbimazole	9	20.9
Current thyroid status (biochemical, TFT)	Euthyroid	28	54.9
	Hyperthyroid	17	33.3
	Hypothyroid	6	11.8
Current thyroid status (clinical, Wayne)	Hyperthyroid	7	13.7
	Euthyroid	36	70.6
	Equivocal	8	15.7

ATD = anti-thyroid drugs, IQR = inter-quartile ratio, TMNG = toxic multinodular goiter, TA = toxic adenoma, TFT = thyroid function test.

* Remission- 6, ATD discontinued for adverse effects- 6.

### 3.2. Prevalence of secondary osteoporosis due to hyperthyroidism

**Figure 1.**
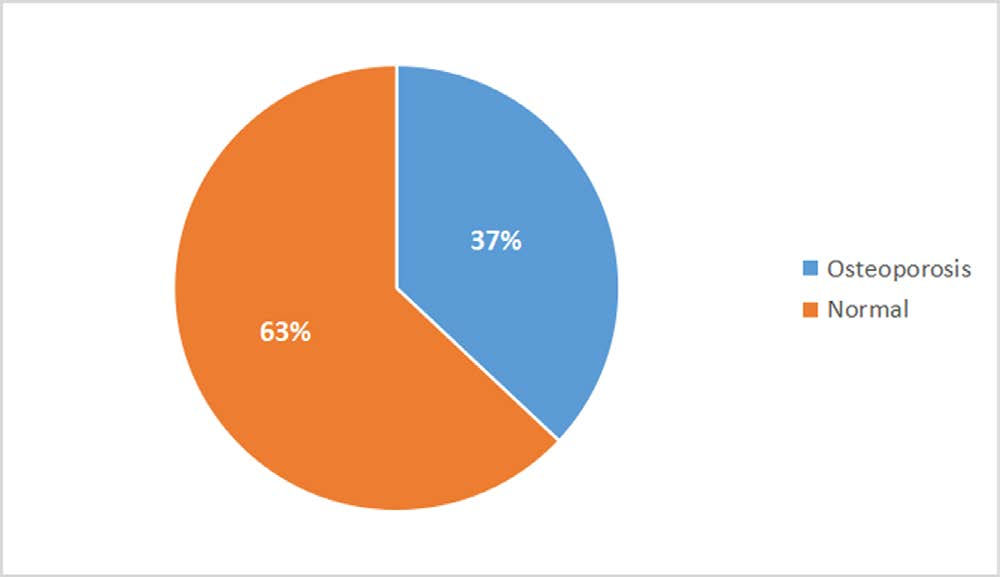
Prevalence of osteoporosis among the study participants (n = 51).

**Figure 2.**
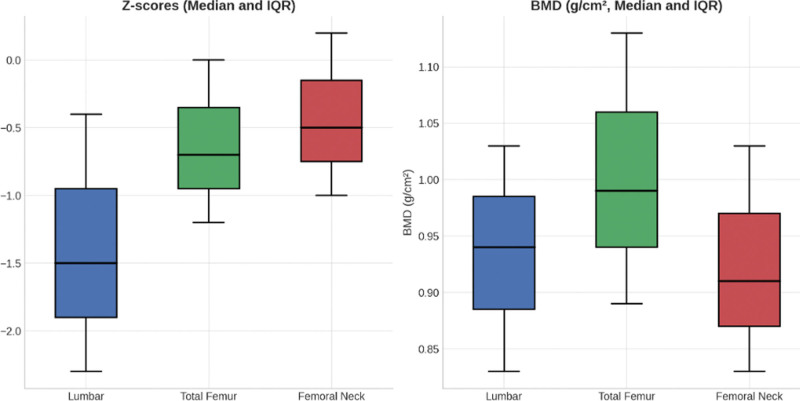
Median Z-scores and BMD with inter-quartile ranges at the lumbar spine, total hip, and femoral neck in study participants (n = 51). BMD = bone mineral density.

More than a third 19 (37%) of the participants had osteoporosis (Z-scores <−2.0) (Fig. 1). For the total study population, the median (IQR) Z-score for lumbar, left total hip and left femoral neck are −1.5 (−2.3, −0.4), −0.7 (−1.2, 0), and −0.5 (−1, 0.2), respectively. Similarly, the median (IQR) BMD for lumbar, left total hip, and left femoral neck are 0.94 (0.83, 1.03), 0.99 (0.89, 1.13), and 0.91 (0.83, 1.03), respectively (Fig. 2).

### 3.3. Factors associated with secondary osteoporosis due to hyperthyroidism

Though, on bivariate analysis, lower body mass index, biochemical and clinical hyperthyroidism (thyroid stimulating hormone, Free T4), high PTU dose and longer duration of hyperthyroidism showed trends toward higher rates of osteoporosis, no single factor came out as an independent predictor of osteoporosis risk in our hyperthyroid patients with no traditional risk factors (Table 3). There was no difference between patients with subclinical hyperthyroidism in comparison to overt hyperthyroidism.

**Table 3:** Factors associated with secondary osteoporosis due to hyperthyroidism in study population.

Variable		Osteoporosis n (%)		COR (95% CI)	AOR (95% CI)	*P*
		No	Yes			
BMI (kg/m^2^)	<25	11 (34.3)	10 (52.6)	2.1 (0.6–6.7)	0.33 (0.07–1.49)	.15
	>25	21 (65.7)	9 (47.4)	1	–	–
Wayne score	Euthyroid	24 (75)	12 (63.2)	1.75 (0.5–5.98)	0.4 (0.59–2.8)	.36
	Hyperthyroid	8 (25)	7 (36.8)	1	–	–
TFT	Hyperthyroid	3 (10)	6 (31.6)	1.8 (0.9–3.6)	0.2 (0.014–3.3)	.27
	Euthyroid	25 (78)	11 (57.9)	1	–	–
	Hypothyroid	4 (12)	2 (10.5)	1	–	–
PTU dose	<300	16 (80)	12 (85.7)	1.5 (0.24–9.6)	0.6 (0.073–4.7)	.6
	>300	4 (20)	2 (14.3)	1	–	–
Duration of hyperthyroidism	<5 yr	18 (56.25)	11 (57.9)	1.07 (0.34–3.4)	0.7 (0.14–4)	.7
	>5 yr	14 (43.75)	8 (42.1)	1	–	–

AOR = adjusted odds ratio, BMI = body mass index, COR = crude odds ratio, TFT = thyroid function test, PTU = propylthiouracil.

## 4. Discussion

This study demonstrated that over one-third (37.3%) of patients with hyperthyroidism who lacked traditional osteoporosis risk factors had DXA-confirmed low bone density (BMD), consistent with secondary osteoporosis. This underscores the significant skeletal impact of hyperthyroidism even in relatively young and otherwise low risk individuals. The findings reinforce that excess thyroid hormone, independent of age, sex, or menopausal status, exerts a deleterious effect on bone metabolism.^[13,14]^

The observed prevalence aligns with international data but is somewhat lower than the 45% reported in the Nigerian cohort^[5]^ and higher than the 29% reported in an Indian population.^[13]^ This might be explained by methodological differences utilized. Our study employed lumbar spine and hip DXA, which are more robust indicators of overall bone mass while prior studies relied on distal radius measurements that primarily reflect cortical bone loss.^[5]^ More importantly, our study excluded post-menopausal women, older adults and patients with other traditional risk factors for accelerated bone loss in an attempt to observe the impact of excess thyroid hormones independently. This might also explain the higher median Z-score (−1.5) compared to prior studies.^[15]^

Consistent with previous studies, we found no significant association between osteoporosis and patient age, sex, or duration of hyperthyroidism.^[5,15]^ This reinforces that thyroid hormone excess is a dominant, independent determinant of bone loss across demographic subgroups. This is particularly relevant to African populations, screening for bone health is often limited to post-menopausal women.

Uncontrolled hyperthyroidism showed a clear trend towards increased risk of osteoporosis, though statistical significance was not reached. This finding mirrors previous studies reporting a strong association between severity and duration of thyrotoxicosis and low BMD^[5]^ while other studies failed to show the difference.^[16]^ Nonetheless, this trend suggests that optimal biochemical control may play a central role mitigating bone loss, emphasizing the importance of regular monitoring and dose titration of ATDs since hyperthyroidism significantly increases fracture risk.^[16]^

Our results contribute novel regional evidence, being the first Ethiopian study to evaluate BMD in hyperthyroid patients using DXA. The findings highlight that even in the absence of conventional risk factors, such as menopause, glucocorticoids use or smoking, bone loss is clinically significant concern. This observation is particularly important in sub-Saharan African context, where both hyperthyroidism and osteoporosis are underdiagnosed and under-screened due to limited diagnostic infrastructure. The study, thus, underscores an unmet clinical need for integrating routine BMD assessment into the management algorithm for hyperthyroid patients.

Though our study rigorously excluded confounding causes of osteoporosis, focused mainly on relatively younger population and used gold-standard DXA scanning, it had a few limitations. The single center cross-sectional design and the modest sample size limits causal inference. The findings could not be compared with the general Ethiopian population due to lack of population-based studies. Additionally, we relied on alkaline phosphatase and other bone turnover markers (such as osteocalcin or C-terminal telopeptide of type I collagen [CTX]) were not assessed. Despite these constraints, the study provides essential groundwork for future longitudinal and interventional research in African settings.

## Acknowledgments

We would like to thank the Department of Internal Medicine of Addis Ababa University, data collectors and study participants for their time and cooperation.

## Author contributions

**Conceptualization:** Mahlet Tadesse.

**Data curation:** Mahlet Tadesse, Brook Alemayehu Tesfaye, Bemnet Taye.

**Formal analysis:** Mahlet Tadesse, Ahmed Reja.

**Funding acquisition:** Mahlet Tadesse, Brook Alemayehu Tesfaye.

**Methodology:** Ahmed Reja.

**Writing – original draft:** Mahlet Tadesse.

**Writing – review & editing:** Mahlet Tadesse, Brook Alemayehu Tesfaye, Bemnet Taye, Meti Wole, Mihiret Gebre, Sisaynesh Angota, Ahmed Reja.


