# Supplementary material for: Osteoporosis independent of traditional risk factors in hyperthyroid patients in a resource-limited setting: An observational study
Source: Medicine (Baltimore). 2026 May 8;105(19):e48715. doi: 10.1097/MD.0000000000048715 (PMC13166608; doi:10.1097/MD.0000000000048715)
Supplement: Supplementary file 1 [file medi-105-e48715-s001.docx]

# SUPPLEMENTARY DOCUMENT

##

## Operational definition

**Osteoporosis** - with radiological evidence of osteoporosis either using the International Society of Clinical Densitometry (ISCD) criteria (established (severe) osteoporosis *z*-score < −2.0 with one or two osteoporotic fractures or osteoporosis by *z*-score < −2.0). The present study utilized the International Society of Clinical Densitometry criteria as the patients fall within the young age group (15).

**Hyperthyroidism** - is defined by clinical and/or biochemical evidence of hyperthyroidism: FT4 > 22.0 pmol/L, FT3 > 6.5 pmol/L, and TSH < 0.3 *μ*/U/ml. Overt hyperthyroidism is defined as Wayne score > 19 in addition to the biochemical pointers of thyrotoxicosis (63).

**Current Alcohol takers**: Individuals who have consumed at least one standard drink in the past 12 months

**Alcohol non-takers**: Individuals who have consumed zero standard drinks in the past 12 months

**Tea and Coffee takers**: Individuals who consume at least one cup per week

**Tea and Coffee non-takers:** Individuals who consume less than 1 cup per week or have not consumed the beverage in the last 30 days.

**Physical Exercise**: Individuals who meet the minimum threshold of 150 minutes of moderate-intensity activity per week.

**Increased bone turnover** is present if: total alkaline phosphatase > 130 U/L (bone formation marker) (15).

**Overt hyperthyroidism**: - is a condition where the body has high levels of thyroid hormones. It's characterized by low levels of thyroid-stimulating hormone (TSH) and high levels of triiodothyronine (T3) and/or free thyroxine (fT4).

**Subclinical hyperthyroidism:** -defined as TSH outside the reference range of values and FT3 and FT4 within the normal range (8).. (17).

**Secondary osteoporosis-**  is defined osteoporosis that occurs as a result of a medical condition or medication~~.~~ (17).

**FRAX score** - a Fracture Risk Assessment Tool that estimates the 10-year probability of hip fracture and major osteoporotic fracture (hip, clinical spine, proximal humerus, or forearm) for **untreated** patients from age 40 to 90 years (64–66).

**DXA scan** - DXA measures bone mineral content (BMC, in grams) and bone area (BA, in square centimeters), then calculates "areal" BMD (aBMD) in g/cm^2^ by dividing BMC by BA (7,37,67)

**Adherent to anti thyroid drugs:** respondents who scored ≥ 6 points of the Morisky medication adherence scale (68).

**Smoking status:** Participants were classified as current smokers if they smoked at least one cigarette per day, as occasional smokers if they smoked usually less than one cigarette per day, as former smokers if they had smoked regularly or occasionally in the past and as never smokers if they had never smoked (69).

**Body mass index: -** was calculated from measured height and weight as weight in kilograms divided by height in meters squared (62).

**Hyperuricemia** was defined as a serum uric acid concentration ≥ 7.0 mg/dL in men and ≥ 6 mg/dL in women (37).
